# Supplementary material for: Chromosomal toxin-antitoxin systems in Pseudomonas putida are rather selfish than beneficial
Source: Sci Rep. 2020 Jun 8;10:9230. doi: 10.1038/s41598-020-65504-0 (PMC7280312; doi:10.1038/s41598-020-65504-0)
Supplement: Supplementary file 1 — Supplementary Figure S1. [file 41598_2020_65504_MOESM1_ESM.pdf]

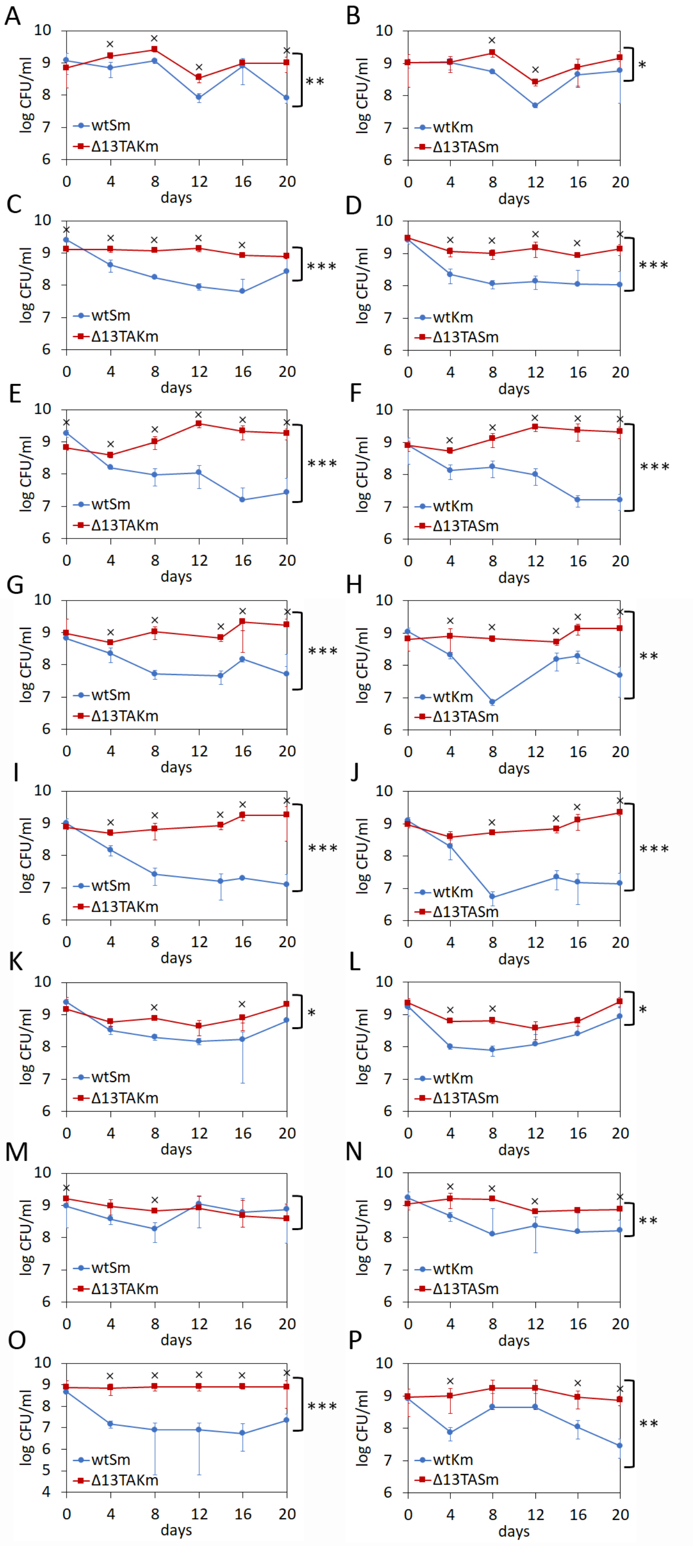

Supplementary Figure S1. 13 TA systems can decrease the competitive fitness of *P. putida* under optimal growth conditions. Co-cultivation of *P. putida* wild-type and Δ13TA strains that are marked with an antibiotic resistance gene (streptomycin or kanamycin). Bacteria were grown in LB medium at 30 °C, diluted into a fresh LB medium every 2 days and CFU/ml was measured on indicated days. Means from four parallels of one measurement with standard deviation are presented. Two-way ANOVA test was used to evaluate the difference between the two strains over all time points (*, P<0.01; **, P<0.0001; ***, P<0.000001). Wilcoxon rank-sum test was used to evaluate the difference in CFU between the strains in each time point separately (x, P<0.05).
