# Supplementary figures and images for: Chromosomal toxin-antitoxin systems in Pseudomonas putida are rather selfish than beneficial
Source: Sci Rep. 2020 Jun 8;10:9230. doi: 10.1038/s41598-020-65504-0 (PMC7280312; doi:10.1038/s41598-020-65504-0)

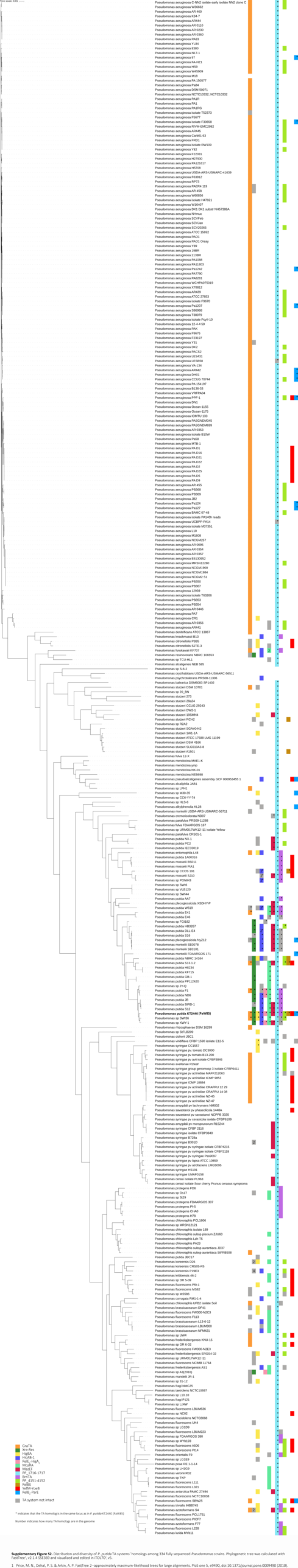

Supplement: Supplementary file 2 — Supplementary Figure S2. [file 41598_2020_65504_MOESM2_ESM.pdf]
